# Contemporary Outcomes after Transurethral Procedures for Bladder Neck Contracture Following Endoscopic Treatment of Benign Prostatic Hyperplasia

**DOI:** 10.3390/jcm10132884

**Published:** 2021-06-29

**Authors:** Clemens M. Rosenbaum, Malte W. Vetterlein, Margit Fisch, Philipp Reiss, Thomas Stefan Worst, Jennifer Kranz, Joachim Steffens, Luis A. Kluth, Daniel Pfalzgraf, on behalf of the Trauma and Reconstructive Urology Working Party of the European Association of Urology (EAU) Young Academic Urologists (YAU)

**Affiliations:** 1 Department of Urology, University Medical Center Hamburg-Eppendorf, 20251 Hamburg, Germany; rosenbaumclemens@gmail.com (C.M.R.); m.vetterlein@uke.de (M.W.V.); m.fisch@uke.de (M.F.); c.philip.reiss@googlemail.com (P.R.); 2 Department of Urology, Asklepios Hospital Hamburg Barmbek, 22307 Hamburg, Germany; 3 Department of Urology, University Medical Center Mannheim, Heidelberg University, 68167 Mannheim, Germany; Thomas.Worst@medma.uni-heidelberg.de (T.S.W.); Daniel.Pfalzgraf@artemed.de (D.P.); 4 Department of Urology, St.-Antonius-Hospital, 52249 Eschweiler, Germany; jennifer.kranz@sah-eschweiler.de (J.K.); joachim.steffens@sah-eschweiler.de (J.S.); 5 Department of Urology, University Medical Center Halle, 06120 Halle, Germany; 6 Department of Urology, University Hospital, Goethe University Frankfurt am Main, 60590 Frankfurt, Germany; 7 Department of Urology, Heilig-Geist-Hospital, 64625 Bensheim, Germany

**Keywords:** bladder neck stenosis, BPH, HoLEP, TURP, endourology

## Abstract

Objectives: Bladder neck contracture (BNC) is a bothersome complication following endoscopic treatment for benign prostatic hyperplasia (BPH). The objective of our study was to give a more realistic insight into contemporary endoscopic BNC treatment and to evaluate and identify risk factors associated with inferior outcome. Material and Methods: We identified patients who underwent transurethral treatment for BNC secondary to previous endoscopic therapy for BPH between March 2009 and October 2016. Patients with vesico-urethral anastomotic stenosis after radical prostatectomy were excluded. Digital charts were reviewed for re-admissions and re-visits at our institutions and patients were contacted personally for follow-up. Our non-validated questionnaire assessed previous urologic therapies (including radiotherapy, endoscopic, and open surgery), time to eventual further therapy in case of BNC recurrence, and the modality of recurrence management. Results: Of 60 patients, 49 (82%) and 11 (18%) underwent transurethral bladder neck resection and incision, respectively. Initial BPH therapy was transurethral resection of the prostate (TURP) in 54 (90%) and holmium laser enucleation of the prostate (HoLEP) in six (10%) patients. Median time from prior therapy was 8.5 (IQR 5.3–14) months and differed significantly in those with (6.5 months; IQR 4–10) and those without BNC recurrence (10 months; IQR 6–20; *p* = 0.046). Thirty-three patients (55%) underwent initial endoscopic treatment, and 27 (45%) repeated endoscopic treatment for BNC. In initially-treated patients, time since BPH surgery differed significantly between those with a recurrence (median 7.5 months; IQR 6–9) compared to those treated successfully (median 12 months; IQR 9–25; *p* = 0.01). In patients with repeated treatment, median time from prior BNC therapy did not differ between those with (4.5 months; IQR 2–12) and those without a recurrence (6 months; IQR 6–10; *p* = 0.6). Overall, BNC treatment was successful in 32 patients (53%). The observed success rate of BNC treatment was significantly higher after HoLEP compared to TURP (100% vs. 48%; *p* = 0.026). Type of BNC treatment, number of BNC treatment, and age at surgery did not influence the outcome. Conclusions: A longer time interval between previous BPH therapy and subsequent BNC incidence seems to favorably affect treatment success of endoscopic BNC treatment, and transurethral resection and incision appear equally effective. Granted the relatively small sample size, BNC treatment success seems to be higher after HoLEP compared to TURP, which warrants validation in larger cohorts.

## 1. Introduction

Bladder neck contracture (BNC) is a bothersome complication following endoscopic treatment for benign prostatic hyperplasia (BPH). The incidence varies between different treatment modalities and is reported with 0.3–9.2% after transurethral resection of the prostate (TURP) [1,2], with no significant difference between monopolar and bipolar resection [3]. After holmium or thulium laser enucleation of the prostate, incidence rates of 2% (HoLEP) [4] and 2.2% (ThuLEP) [5] are reported.

Considering the number of endoscopic treatments for BPH with approximately 74,980 procedures in 2012 in Germany (according to data from the Federal Statistical Office of Germany) and approximately 101,195 procedures in 2008 in the US (among Medicare patients) [6], the importance of the subject becomes obvious. Considering a BNC incidence of 5% [7], this translates into nearly 4000 patients in Germany and more than 5000 patients in the US suffering from BNC every year.

Numerous techniques of BNC treatment are reported in the literature. In general, they can be divided into endoscopic and open procedures. Among endoscopic treatments, transurethral incision and resection are most common. However, up till now, there is no clear treatment algorithm for BNC and there are no studies to compare the techniques.

Generally, results of BNC and vesico-urethral anastomotic stenosis (VUAS) treatment are reported together. Given the entirely different etiology, anatomy, and outcomes of both entities, this approach should be reassessed. The clinical significance of such distinction between BNC and VUAS was recently underlined with significantly deviating success rates of 30% vs. 53% after treatment for VUAS and BNC, respectively [8].

Another drawback in the literature is that the available evidence is mainly based on single-institution data and, given small sample sizes, the impossibility to identify potential risk factors for treatment failure after BNC treatment.

Against this backdrop, we performed a multi-institutional evaluation of patients only undergoing endoscopic treatment for BNC. We aimed to (1) give a more realistic, real life insight into contemporary BNC treatment and results, and (2) evaluate and identify risk factors associated with inferior outcome of BNC treatment.

## 2. Material and Methods

### 2.1. Study Population

After obtaining Institutional Review Board approvals (ethical approval number PV5205), all patients who underwent transurethral treatment for BNC between March 2009 and October 2016 were retrospectively extracted from three institutional databases (University Medical Center Hamburg-Eppendorf, Hamburg, Germany; St.-Antonius-Hospital, Eschweiler, Germany; University Medical Center Mannheim, Mannheim, Germany; Heidelberg University, Heidelberg, Germany) and subsequently merged into a multicenter dataset. As mentioned above, we aimed to only analyze BNC patients. Therefore, no VUAS patients were included. As Pansadoro et al. described BNC to be different after simple prostatectomy compared to transurethral procedures [9], we excluded one patient with previous simple prostatectomy to avoid potential bias. Further, two patients were lost to follow-up (FU) and were excluded prior to final analyses.

### 2.2. Covariates and Definition of Disease Recurrence

Digital charts were reviewed for clinical information and re-admissions or re-visits to our institutions. Patient and surgical characteristics were assessed and included age, institution, type of previous BPH treatment (TURP vs. HoLEP), number of previous interventions for BNC, time from any previous endoscopic treatment, time from previous BPH treatment, and time from previous intervention for BNC (if applicable). Further, patients were stratified by the type of current BNC treatment technique (transurethral resection of bladder neck (TUR-BN) vs. transurethral incision of bladder neck (TUI-BN)).

Patients were contacted personally for FU and interviewed for the occurrence of de novo incontinence (yes vs. no), recurrence status, and recurrence management.

Disease recurrence was defined as any need for further instrumentation such as catheterization, dilation, internal urethrotomy, or open surgery. Diagnosis of recurrence was mainly patient driven due to recurrent lower urinary tract symptoms. There was no standardized follow-up at our institutions.

### 2.3. Surgical Techniques

The applied principle was to widen the bladder neck up to 26 F. The transurethral armamentarium included TUR-BN, a circular 360° resection of the BNC, as well as TUI-BN, by which the incisions were carried out at 4, 8, and 12 o’clock in lithotomy position. Due to the retrospective character of this analysis, type of intervention was left to surgeon’s preferences.

### 2.4. Statistical Analyses

The primary outcome was the success rate after different types of endoscopic procedures. The secondary goal was to identify risk factors influencing the outcome. To achieve that, patients were stratified according to recurrence status (no recurrence vs. disease recurrence) and patient and surgical characteristics were compared between the groups by descriptive statistics. The distribution of categorical variables was reported by frequencies and proportions. Means, standard deviation (SDs), medians, and interquartile ranges (IQRs) were reported for continuously coded variables depending on whether they were normally distributed. Chi-squared tests, Fisher’s exact test, and *t*-tests were used to model the differences between the groups, as appropriate.

All statistical analyses were performed using Stata^®^ (StataCorp. 2015. Stata Statistical Software: Release 14. College Station, TX: StataCorp LP). The reported *P* values were two-sided and values < 0.05 were considered statistically significant.

## 3. Results

We identified 60 patients who underwent endoscopic therapy for BNC. Of those 60 patients, 44 (73%), 10 (17%), and 6 (10%) were operated in the participating institutions. Demographic data are shown in Table 1. Mean age of the entire cohort was 69 (±8.8) years. Overall, 49 (82%) underwent TUR-BN and 11 (18%) underwent TUI-BN. Previous BPH treatment was TURP in 54 (90%) patients and HoLEP in 6 (10%) patients.

Median time from any previous therapy was 8.5 months (IQR 5–14). Median time from prior therapy differed significantly in those with (6.5 (IQR 4–10) months) and those without a recurrence of the BNC (10 (6–20) months; *p* = 0.046).

Median FU was 22 months (IQR 12–32). Overall, BNC treatment was successful in 32 patients (53%). Whereas none of the patients with previous HoLEP (0 of 6) suffered from disease recurrence after endoscopic BNC treatment at median FU, there were BNC recurrences in 52% of patients (28 of 54) who had previously undergone TURP for BPH (*p* = 0.026). Further, there were no differences across the groups regarding age, type of current BNC treatment, or number of previous treatments (all *p* > 0.1; Table 1).

Of the patients, 33 patients (55%) underwent initial endoscopic treatment, and 27 patients (45%) underwent repeated endoscopic treatment for BNC. In initially treated patients, median time from previous BPH treatment was 9 (IQR 6–20) months. In those patients, time from previous BPH treatment differed significantly in those with a recurrence (7.5 (IQR 6–9) months) compared to those treated successfully (12 (IQR 9–25) months; *p* = 0.01). In patients with repeated treatment, median time from previous intervention for BNC was 6 (IQR 4–11) months. In these patients, median time from previous intervention for BNC did not differ in those with (4.5 (IQR 2–12) months) and without a recurrence (6 (IQR 6–10) months; *p* = 0.6; Table 1).

One patient (1.7%) developed a de novo incontinence postoperatively, defined according to patient supplied information. Radiation therapy was performed in four patients (6.7%). Radiotherapy did not influence the outcome.

## 4. Discussion

BNC is a common complication of endoscopic treatment for benign prostatic hyperplasia (BPH). Incidence varies between different treatment modalities and ranges from 0–10% [1,2,3,4,5].

As the number of endoscopic treatments for BPH with approximately 74,980 procedures in 2012 in Germany (according to data from the Federal Statistical Office of Germany) and approximately 101,195 procedures in 2008 in the US (among Medicare patients) [6] is high, the importance of the subject is obvious.

While some risk factors for the development of BNC have been established or proposed, apparently, little can be done to safely avoid them. Established risk factors are small prostatic size at surgery [8,9], untreated prostatitis at time of surgery, long resection time, and extensive resection at the bladder outlet [10].

Numerous techniques of BNC treatment are reported in the literature. In general, they can be divided into endoscopic and open procedures. Among endoscopic treatments, dilations have been used as well as incisions and resections with varying outcome. More recently, incision followed by injection of mitomycin C (MMC) [10] was reported. In open surgery, YV-plasty or T-plasty can be useful for recurrent or recalcitrant cases [11,12,13].

While BNC and VUAS are commonly reported together, their etiologies are entirely different. Consequently, this study focuses on patients with BNC only. As for VUAS, where multiple therapeutic algorithms exist [14], there is no clear treatment algorithm for BNC, nor are there randomized studies to compare the techniques. Rather, single techniques are explored in multiple studies with varying results. 

Similar to VUAS, where multiple therapeutic algorithms exist side by side, no generally agreed therapeutic scheme exists for BNC. However, endoscopic treatments are preferred as primary treatments, followed by open reconstruction in recurrent cases. As various endoscopic techniques are available and none have been compared directly to each other so far, we decided to retrospectively review the two techniques applied at our centers: TUI-BN and TUR-BN.

The overall success rate for BNC treatment was 53% (32 patients). The success rate after TUR-BN was 55%, after TI 46%, the difference was not statistically significant (*p* = 0.7). Pansadoro and Emiliozzi reported a success rate of 86% for TUI-BN and 65–93% for TUR-BN in treatment-naïve patients with BNC, and while in patients treated with TUI-BN success rate could even be raised with one further treatment, in patients with severe BNC treated by TUR-BN, further interventions did not improve outcome [9]. In our study, type of BNC treatment, number of BNC treatments, and age at treatment did not differ between patients without vs. with recurrence. Again, small sample size possibly influences results and larger cohorts will have to confirm our results. Further, comparing TUR-BN and TUI-BN can inherit a potential bias as patients with milder BNC were potentially treated with TUI-BN and those with severe BNC-treated TUR-BN. 

In refractory cases, reconstructive techniques like open or robot-assisted laparoscopic YV-plasty [7,13] or its modification, the T-plasty [11,12], can achieve good results. Recently, robot-assisted laparoscopic subtrigonal inlay of buccal mucosa for recurrent BNC was reported [15]. In our algorithm, open reconstruction is performed latest after three attempts of endoscopic treatment, as Kranz et al. [8] showed a significantly lower success rate for endoscopic treatments if performed more often.

In patients not previously treated for BNC, time since BPH surgery to BNC treatment was significantly shorter in those with a recurrence compared to those treated successfully (7.5 vs. 12 months; *p* = 0.01). The reason for the earlier onset of BNC in patients tending to a recurrence might be differences on a molecular basis. Similarly, we found differences in scar tissue from VUAS after radical prostatectomy between patients successfully treated for VUAS and those with recurrences [16]. Data from this study suggest that transcriptomic changes can serve as diagnostic markers [17], possibly allowing for better risk stratification in the future [18]. Future studies with tissue samples from BNC could shed light on possible transcriptomic patterns in the tissue of the bladder neck, predetermining development of BNC. In patients with repeated treatment, median time from prior BNC therapy did not differ in those with or without further recurrences.

Success rate of BNC treatment was significantly higher if BPH treatment was HoLEP compared to TURP (100% vs. 48%; *p* = 0.026). The reason for this remains unclear. A possible explanation is more extensive coagulation at the bladder neck in TURP patients and the smaller prostate size in TURP patients as compared to HoLEP. However, the statistical relevance has to be judged in the light of the different sample sizes between the HoLEP and TURP groups. Moreover, statistically relevant difference is reached quickly in very small groups such as ours. Results will have to be confirmed in larger cohorts. 

De novo-incontinence occurs rarely after BNC treatment. This is probably due to the typical location of the stricture. As only few strictures reach close to the sphincter, this might explain the low risk to affect the sphincter.

Despite the multi-institutional character of our study including 60 patients with BNC, our findings have to be interpreted as hypothesis-generating due to the still relatively small sample size with low number of events, which did not allow for performance of multivariable analyses to identify true associations between independent variables and the endpoint recurrence. Moreover, the definition of recurrence as any further instrumentation is likely to underestimate the true recurrence rate. On the other hand, potential other lower urinary tract disorders (e.g., urethral stricture or atonic bladder) may lead to an untrue diagnosis of BNC recurrence. Therefore, cystoscopic outcome measures in a prospective way are needed. Other limitations are the short-term follow-up with potential underestimation of recurrences and the use of non-validated questionnaires. The observed differences (e.g., results regarding BNC treatment efficacy after TURP vs. HoLEP or the comparison of TUR-BN and TUI-BN) are based on descriptive analyses: Thus, future prospective assessment with larger samples are needed to validate this hypothesis.

## 5. Conclusions

A longer time interval between previous BPH therapy and subsequent BNC incidence seems to favorably affect treatment success of endoscopic BNC treatment. In our small cohort, transurethral resection and incision appear equally effective. Granted the relatively small sample size, BNC treatment success seems to be higher after HoLEP compared to TURP, which warrants validation in larger cohorts.

## Figures and Tables

**Table 1:** Patient characteristics of 60 men who underwent endoscopic treatment for bladder neck contracture stratified by recurrence status.

	Overall	No Recurrence	Recurrence	*p* Value
Number of patients; *n* (%)	60 (100)	32 (53)	28 (47)	---
Age (years); mean ± SD	69 ± 8.8	68 ± 9.4	70 ± 8.2	0.4
Previous BPH treatment; *n* (%)				0.026
TURP	54 (90)	26 (48)	28 (52)	
HoLEP	6 (10)	6 (100)	0 (0)	
Number of previous treatments; *n* (%)				0.5
0	33 (55)	19 (58)	14 (42)	
≥1	27 (45)	13 (48)	14 (52)	
Time from any previous endoscopic treatment (months); median (IQR)	8.5 (5–14)	10 (6–20)	6.5 (4–10)	0.046
Time from previous BPH treatment (months); median (IQR)	9 (6–20)	12 (9–25)	7.5 (6–9)	0.010
Time from previous intervention for BNC (months); median (IQR)	6 (4–11)	6 (6–10)	4.5 (2–12)	0.6
Type of current BNC treatment; *n* (%)				0.7
TUR-BN	49 (82)	27 (55)	22 (45)	
TUI-BN	11 (18)	5 (46)	6 (55)	

BNC, bladder neck contracture; BPH, benign prostatic hyperplasia; HoLEP, holmium laser enucleation of the prostate; IQR, interquartile range; SD, standard deviation; TUR-BN, transurethral resection of bladder neck; TUI-BN, transurethral incision of bladder neck.

## Data Availability

Data available on request.
